# Early Identification of Mental Illness in Primary School Pupils by School Nurses: A Qualitative Study

**DOI:** 10.1177/23779608221081452

**Published:** 2022-02-25

**Authors:** Vedrana Vejzovic, Lilliann Carlson, Lisa Löfgren, Ann-Cathrine Bramhagen

**Affiliations:** 1 5264 Malmö University, Faculty of Health and Society, Department of Care Science, Malmö, Sweden; 2 Department of Care Science, Specialist nurse Malmö University, Faculty of Health and Society, Malmö, Sweden; 3 Faculty of Health and Society, Department of Care Science, School nurse, Malmö University, Malmö, Sweden; 4 Faculty of Health and Society, Department of Care Science, 5264 Malmö University, Malmö, Sweden

**Keywords:** guidance, mental illness, pupils, school nurses

## Abstract

**Introduction:**

The World Health Organization has reported that one fifth of all children in the world suffer from poor mental health regardless of cultural differences. Previous studies have shown that working with mental health is an important part of the duties of school nurses in Sweden.

**Objective:**

The aim of the present study was to describe the experiences of school nurses regarding the identification of mental illness among pupils in primary school.

**Methods:**

In this inductive qualitative study, interviews were conducted with 11 school nurses in southern Sweden and analyzed using content analysis.

**Results:**

The results indicate three major themes: (1) the need for shared responsibility, (2) feelings of uncertainty and inadequacy in school nurses, and (3) the importance of establishing relationships.

**Conclusion:**

This study indicates that school nurses feel responsible for their pupils’ well-being, but also feel that they need support. A lack of guidance in identifying mental illness emerged from the interviews.

## Introduction

The incidence of mental health issues in the pediatric population, the role of the school nurse in identifying issues connected with mental health in pupils, and the knowledge or feelings school nurses have about this role are all different topics than will be presented. According to a report by the World Health Organization (WHO, 2019), a fifth of all children in the world suffer from poorer mental health that manifest in similar ways regardless of cultural differences. Furthermore, the report points out that there are global injustices with regard to access to effective, professional help for them who suffering from poorer mental health, and that this is not in line with Chapter 3 of the UN Convention on the Rights of the Child (1989), which states that the best interests of the child should always be prioritized. Mental illness is as an umbrella term that covers short-term psychological and emotional distress, mild anxiety, mild depression, and other symptoms that meet the guidelines for a psychiatric diagnosis lasting for at least two weeks (WHO, 2008).

### Review of Literature

Previous studies have shown that identifying children who display early indicators of potential psychological problems and providing supportive therapeutic interventions to those with a diagnosed mental illness are important components of the work of a school nurse (Ellertsson et al., 2017; Garmy et al., 2015). Moreover, the general perception of school nurses is that they play a central role in supporting children with mental illness (Ravenna & Cleaver, 2016; Jönsson et al., 2019). The overall mission of the school nurse is to promote pupils’ learning development and health, which should take into account factors that impact well-being or contribute to learning difficulties. This can be achieved by offering competent nursing services, effective support, and identifying problems or symptoms at an early stage during health visits,. School nurses are also responsible for physical examinations such as weight, height, and for vaccinations (The National Board of Health and Welfare, 2016).

The obstacles encountered by school nurses in providing care for children with mental illness include time constraints due to a heavy workload, limited education specific to mental illness in children, and low levels of confidence in their ability to respond effectively. School nurses were aware of personal and professional development opportunities to address their limited education and confidence; however, they also consider the work of supporting mental health and well-being in children as an opportunity for personal and professional development (Jönsson et al., 2019). Early discovery and treatment of mental illness has proved to be highly beneficial for affected pupils, as it reduces the risk of negative consequences of long-lasting mental illness (Allison et al., 2014). However, Doi et al. (2018) found that school nurses felt ill-equipped to work effectively with children exhibiting signs and symptoms indicative of mild to moderate psychological and emotional distress and illness. Turner and Mackay (2015) reported that school staff were unsure regarding whether schools were a suitable place for interventions designed to prevent mental illness. According to the existing literature, good communication between professionals who provide mutual advice and support helped improve both professional relationships and the care provided to pupils (Turner & Mackay, 2015).

Dina and Pajalic (2014) emphasized the importance of working with caregivers, with the whole family, when a school child is experiencing mental illness. Conversations with pupils regarding health and well-being have proved helpful in identifying emerging mental illness when a child presents repeatedly for unscheduled nurse’s visits with somatic symptoms such as headaches or pain in other parts of the body (Dina & Pajalic, 2014). With regard to spontaneous visits to the school nurse, studies have shown that schoolgirls report mental illness more often than boys (Hutton et al., 2014), and that girls also seek help from the school nurses more often than boys (Ellertsson et al., 2017).

Studies indicate that mental illness among both children and adolescents is increasing (Bremberg, 2015; Collishaw, 2015; WHO, 2019), resulting in adverse consequences for the child or adolescent and for the wider community (Public Health Agency of Sweden, 2018). School nurses in Sweden meet almost all children and adolescents, as their profession involves interacting with all pupils who attend school, and this gives them an opportunity to identify emerging mental illness among children and adolescents at an early stage (Jönsson et al., 2019). Garmy et al. (2015) investigated programs designed to prevent mental illness initiated by experienced school nurses, as being valuable and meaningful by the pupils. However, research exploring the experiences of school nurses in detecting mental illness is limited (Membride et al., 2015). Given the evidence of increasing mental illness among children and adolescents, it is important to examine the experiences of school nurses in this area. Thus, the aim of the present study was to describe the experiences of school nurses regarding the identification of mental illness among pupils in primary school.

## Methods

This study employed a descriptive qualitative methodology using semi-structured interviews for data collection and qualitative content analysis for data analysis (Burnard et al., 2008). The study was reported according to O'Brien et al.’s (2014) Standards for Reporting Qualitative Research (SRQR).

According to Swedish law, interviewing professionals regarding their work does not require approval from the Swedish Ethical Review Authority. Prior to commencing the project, the institutional review board reviewed the project proposal, and no changes were requested.

Settings

The study was conducted in public primary schools in two different communities in the county of Skåne in southern Sweden. Primary school is mandatory for children in Sweden aged 6–15 years (during nine years). Each school nurse in this study was responsible for approximately 460 pupils. In order to work with schoolchildren, a school nurse in Sweden needs to specialize at the Master’s level in public health and/or child and adolescent health.

Participants

A total of 11 school nurses participated. The inclusion criteria were registered nurses with a post-graduate education in public health nursing and specialist education in health care for children and adolescents or in school health care, with more than one year of work experience as a school nurse. Five participants were specialists in child and adolescent health, and three in public health. Three school nurses had dual competencies—two were both a public health nurse and a school nurse, and one was a public health nurse and a psychiatric nurse. All participants had worked between 2 and 19 years, with a median of 10 years. One school nurse who had agreed to participate had to cancel her interview due to illness.

Data Collection

Information about the study was forwarded by e-mail to school nurses at each study site after obtaining permission from each principal. Those interested emailed the interviewer and gave their consent to be contacted by telephone for further discussion regarding the time and place for the interviews. The authors were contacted by 12 nurses, with one nurse declining the interview. The interviews were conducted over two months (March–April) in 2019. All interviews commenced with the following two questions “Could you tell us about your experiences of mental illness among the children at your school?”, and “Could you tell us about your work regarding the identification of mental illness?”. Follow-up questions, such as, “Could you elaborate on this?”, “What did you do then?”, “Do you have more experience or examples?”, “What do you mean when you say…?” were asked during all the interviews. All interviews were digitally audio recorded and transcribed, and conducted at the workplaces of each school nurse in accordance with the wishes of the informants. The interviews lasted between 31 and 73 min (median 47 min). All participants provided written informed consent before the start of the interview.

Data Analysis

In this study, a qualitative analysis of the data was conducted using an inductive approach to derive the structure of analysis according to Burnard et al. (2008). Initially, all transcripts were read thoroughly and examined by the authors to familiarize themselves with the data and a sense of the whole. To obtain a summary statement of each element discussed in the transcript, the authors made notes summing up the contents of the text, after which an open coding was performed. In the second stage, all words were collected in another document and worked through to reduce the number of categories. All authors actively participated in the analysis, and finally returned to the transcripts to ensure that no important data were excluded. Through discussion and reflection, three categories were created. The qualitative analysis was based on a close interpretation of the text, which implies manifest content (Burnard et al., 2008). All the authors were pediatric nurses, one of whom was a school nurse. To reduce bias, coding was undertaken; coding was done separately, and codes and categories were discussed within the research group to reach a mutual agreement.

## Results

Three categories emerged from the data analysis of the school nurses’ responses to questions on the detection of mental illness in primary school pupils: (1) the need to share responsibility, (2) feelings of uncertainty and inadequacy, and (3) the importance of relationships.

### The Need to Share Responsibility

The result has shown that school nurses feel responsible yet wish to share the responsibility with teachers and guardians, would like guidelines for this responsibility, and sometimes experience a lack of consensus with teachers and guardians about students. The school nurses affirmed their key role in identifying mental illness as they are the only individuals to meet all the pupils at the school. It was clear that all school nurses felt a strong responsibility for the health status of the pupils and saw themselves as spokespersons for the children, always acting in the interests of the children.

“…I’m here for the sake of the children and as a school nurse you are very much on the side of the children, so to speak.” (Informant 6)

However, this sense of responsibility entailed a feeling of loneliness when coping with the mental illness of their charges. According to the school nurses, increased cooperation within the whole health care team, the teachers, and the principal was desirable in order to deal with mental problems in children. A lack of consensus between the school nurse and other professions was seen as a potential obstacle to health-promoting work. However, the school nurses were also unsure as to who had the main responsibility for identifying indicators of emerging mental illness among pupils.

“It isn’t possible to work in isolation but it’s really, /…/ my part is a small part of a whole.” (Informant 10)

The school nurses also identified a need for clearer guidelines outlining responsibilities in the task of detecting mental illness in children. In situations where a common understanding and shared responsibility existed, the process of identifying mental illness functioned satisfactorily. Some school nurses also highlighted the fact that an important component of the school nurse's role was to provide educational support for the teaching staff, although some felt that the teachers focused too much on pedagogy and less on how the pupils’ health affected their school performance. The school nurses pointed out that although teachers are in closest contact with the pupils, they often miss signs of mental illness. They stressed the importance of having a holistic view of children’s health.

“No offense, but they think about pedagogy and they think in terms of letters and figures and that’s their assignment /…/ then I can feel that maybe that’s not what’s most important now, when you see that someone is, well, not feeling too well.” (Informant 2)

Being in consensus with and cooperating with guardians was also presented as an important part of recognizing mental illness in schoolchildren. The school nurses agreed that the guardians played an important role of the, saying that they had the ultimate responsibility for the child, but noted that mental illness may be stigmatized in certain families. One school nurse raised the issue of the lack of consensus between the school nurse and the guardian and the resulting difficulties.

“…we in the school were the ones who thought he seemed to be in a bad way, the mother didn’t think so and that’s also such a…it’s after all the responsibility of the guardian /…/ we can only express our worry that everything is not as it should be /…/ it’s not altogether simple…” (Informant 2)

### Feelings of Uncertainty and Inadequacy

The school nurses felt uncertain and inadequate in relation to assessment, diagnosis, intervention, and evaluation of children with mental health issues. Study results suggested that participants’ experiences differed in relation to the task of ascertaining mental illness among schoolchildren depending on the age of the children. Further, some participants experienced uncertainty about their own competence. The subject itself was considered complex and delicate, and participants were unsure as to what the concept of mental illness included. They expressed not only feelings of uncertainty regarding their knowledge of the subject but also their own fearfulness about difficult conversations; they also pointed out that children’s body language was sometimes hard to interpret. However, the importance of being honest was emphasized.

“…but it was unpleasant. Then there are of course details and such that also contribute to making me, too…scared. Yes, that’s something one also has to deal with, one’s own fear.” (Informant 7)

“ They say that they think you’re fine and such but based on what we’ve been talking about, I get the impression that maybe things are not quite so good /…/ and just having that confidence, daring to say that…they signal something.” (Informant 1)

Furthermore, the school nurses mentioned that mental illness is a complex subject and children of different ages and girls and boys are differently challenged .

“…they [i.e., boys] don’t come so often but rather tend to isolate themselves /…/ The young ones come very often. The older ones deal with it on their own most of the time, that’s what I think anyway’.” (Informant 4)

School nurses also highlighted the difficulties they faced when attempting to access specialist child and adolescent psychiatry services. Under-resourced services, resulting in long waiting times created further problems and caused frustration. In some cases, this resulted in temporary solutions that were seen as uncertain to succeed. Furthermore, the school nurses clearly indicated that their task was not to treat, although they were aware that it was their responsibility to help pupils.

One sometimes feels frustrated…one knows that there’s a long waiting list for child and adolescent psychiatry…there’s sort of a stop, so that one has to try to, you know, fix things a little, arrange something makeshift…” (Informant 3)

The school nurses felt that they were ill-prepared to identify the indicators of mental illness. One school nurse pointed out that there is an expectation that the school nurse should be competent to detect mental illness, but limited knowledge or access to evidence-based guidelines was seen as a barrier to optimal functioning in this area of school nursing. The lack of clear guidelines in their work often left participants unsure regarding whether they had been effective.

“…how one, well, deals with it /…/ Can I do something more? /…/ I do of course hope that I do things the right way and that the result will be good…but there are other things one could do…” (Informant 9)

It was clear that participants found it easier to assess the physical health of the children than their mental health, and they pointed out that there was information about their pupils’ physical illness in their handbook but nothing about mental illness. Due to the difficulties in assessing and identifying mental illness, they often had to trust their gut feeling. One school nurse described how this feeling was based on a complex assessment of, for example, the pupil’s body language, looks, facial expressions, and behavior.

“…but what I base those feelings on, I don’t know that, I just think it’s…you sort of get a feeling.” (Informant 9)

Participants wished they could identify mental illness earlier.

I think that one maybe does not find the pupils early but instead one usually finds them when they have collapsed and when they are in a really bad way…so I do not really know…how one should have…caught them.” (Informant 3)

Adequate work experience considered to be an important factor in reducing the feeling of inadequacy. Some of the school nurses described that they were less uncertain and inadequate because they were more experienced. Participants described how initially they had rigidly followed the child health program, but how, over time, as they acquired more experience, they had been able to adopt a more flexible approach and were able to interpret signs of mental illness among pupils.

“…one has learned over time /…/ in the beginning one was less alert to it, I think, one didn’t think so much about it, but over time one has learned to see children and interpret, it’s something that’s a result of experience.” (Informant 11)

The majority of the school nurses affirmed that they due to their uncertainty had to seek and received sufficient support from other professions within the healthcare team in the task of identifying mental illness. Although working with mental illness could differ between different schools, the school nurses said that they found support in other colleagues in the same profession with whom they interacted on a regular basis. The quality of this support varied, depending on which colleague they turned to, even though all school nurses have the same assignment.

“…then it also depends a little on what school nurse one talks to…what answer one gets.” (Informant 9)

### Importance of the Relationship Building

Difficulties in a relationship building between a school nurse and the student can lead to late identification of a student's mental illness were presented by school nurses. Some of these speculated that many students hide their true state of health for a long time by keeping a facade and keeping their feelings and thoughts to themselves. In cases where a relationship between nurse and student has not had time to develop, it can be a contributing factor to late identification of mental illness. Due to this late identification, the school nurses speculate about different solutions, but find no simple and successful solution except that they emphasize the importance of having a relationship with the pupils.

Time and accessibility were seen as important prerequisites for relationship building. Participants felt that the lack of time could result in reduced accessibility, and, therefore, reduced opportunities for pupils to make unscheduled visits seeking assistance.

“I’ve been here for so long, you know, and the kids…well, they see me and think it’s great that I’m here and we have a lot of fun together and joke. They feel a security /…/ So I have created a trust capital that I take care not to lose!” (Informant 1)

Some of the school nurses described how they spent time among the pupils more or less daily in order to be always visible and accessible. They talked about how important it was that the pupils knew who they were and that they themselves contributed actively to relationship building*.*

## Discussion

The results that emerged from a qualitative analysis were classified under the following three categories: the need to share responsibility, the feelings of uncertainty and inadequacy, and the importance of relationships. Common categories referred to by all the school nurses interviewed for the study were the need for shared responsibility in detecting mental illness in children, the lack of guidance in effectively discharging this duty, and the lack of clear role descriptions that might be an obstacle to performing this task adequately. The preventive work done by school nurses was self-reported as being deficient. In many cases, the school nurses were guided by emotions during difficult conversations with pupils with signs of mental illness. Similar results have been reported by Jönsson et al. (2019) regarding school nurses’ working with previously identified mental illness among pupils; in that study, school nurses described feeling frustrated at not being sufficiently prepared to support pupils with mental illness, despite their wish to do so.

The results from the present study suggest that participants were uncertain as to how they could identify mental illness due to the lack of clear and structured guidelines. A previous study by Skundberg-Kletthagen and Moen (2017) reported that school nurses asked for more knowledge about inter- and multidisciplinary cooperation regarding the follow-up of pupils with such problems. According to our study results, as school nurses often act based on their own experience and competence, pupils may obtain unequal health care; this is contrary to the Convention on the Rights of the Child, which became law in Sweden in January 2020. In this study, participants asked for more tools and further training; this is in line with reports by Skundberg-Kletthagen and Moen (2017). Improved knowledge of mental health has the potential to increase early intervention, promote mental well-being, and enable effective support of the community (Al-Yateem et al., 2018). With regard to mental health problems and the identification of related problems, our results indicate that teachers act as collaborative partners with school nurses because they meet the adolescents daily and may, therefore, be able to detect changes in mental health, which is in line with the results of Granrud et al.’s study (2019).

In our study, the school nurses stressed that their relationships with their pupils was the cornerstone of their work. They believed that without a trusting relationship, it would not be possible to identify mental illness among pupils, which is consistent with the results of Granrud et al. (2019), who reported the importance of an open-door policy. However, those who spent less time at school found that they were usually fully booked; hence, it was difficult to always be accessible for spontaneous visits from adolescents or teachers (Granrud et al., 2019). This is similar to the experience reported by school nurses in Sweden; the lack of time, combined with the large number of children in the care of each school nurse were considered to be factors that adversely affected the work done by our interviewees. Similar findings were also reported in a study by Skundberg-Kletthagen and Moen (2017), who emphasized the importance of being accessible to the pupils. The lack of accessibility and time was believed to be obstacles to the creation of trusting relationships. Although the school nurses in the present study believed that relationship building was a prerequisite for early identification of mental illness, they struggled with a number of organizational problems that had to be dealt with before they could do their work effectively. Given the challenges clearly emerging from this research, it could be suggested that the task of detecting mental illness may constitute an occupational risk to the health of school nurses. This hypothesis was confirmed by Powell et al. (2018), who showed that a heavy workload, in conjunction with moral dilemmas, created anxiety in school nurses.

Allison et al. (2014) stated that it is common for high-achieving pupils to conceal symptoms of mental illness and that this made it difficult for teachers to perceive warning signs, as these pupils do well in school. The school nurses in this study expressed the need to find a solution that would make it possible to identify problems in these pupils earlier to prevent the development of mental illness later in life. Allison et al. (2014) also highlighted the fact that an important step in the early identification of mental illness is screening by school nurses for anxiety and depression. Garmy et al. (2015) described programs for preventing depressive symptoms in young people. Their study also stressed that, in the future, it is desirable to adopt a more unified approach to mental health issues in pupils, and to include more boys in such programs. However, despite the existence of different preventive interventions, the opportunities for early identification of pupils who need help do not seem to be optimal.

Schulte-Körne (2016) demonstrated the importance of the school, guardians, and healthcare professionals working together in order to detect signs of mental illness as early as possible. We believe that a clearer allocation of responsibilities within the school could contribute to safer and better care for the pupil. This would reduce the workload of the school nurses and ensure that pupils receive timely and effective care. The results of our study confirm that there is still a need for sharing responsibility in the task of identifying mental illness among pupils.

### Strengths and Limitations

The semi-structured interview meant that all the participants could be asked the same questions, which strengthened the dependability of the study*.* Age and work experience varied among interviewees, which can be seen as strength. Overall, study participants had extensive work experience (median work experience of 10 years), which is also an important strength. The material obtained through the 11 interviews was rich and corresponded to the study aims; thus, data collection was considered sufficient, and therefore terminated after the 11 interviews had been conducted. However, it is not known whether new data would have emerged had data collection been allowed to proceed*.* One school nurse canceled her interview due to illness, and it is not possible to know whether that interview would have contributed additional data. Several findings recurred; therefore, we believe that our findings are likely to be shared by other school nurses in Sweden. Regarding credibility, all four authors were pediatric nurses but with different experiences of working with children and with research, and three of the authors were involved in the analysis of the interviews. This can also be seen as part of the confirmability of the study. Efforts have been made to meet the dependability to enable other researchers to repeat our design.

### Implications for Practice

Since a considerable amount of young people suffer from mental illness and the syndrome is similar regardless of cultural differences, school nurses must have a profound knowledge to be able to, firstly identify mental illness among young people and, secondly to handle them correctly. Our study showed that many school nurses felt responsible for the pupil’s mental health but also felt uncertain regarding how, and sometimes who were responsible for these professional challenges. They expressed a need to share the responsibility. So, the implications for practice should be to offer continuous education for the nurses and also give opportunities to receive guidance from for example a psychologist as a support. Furthermore, written guidelines might be one way to support the nurses and to clarity the responsibility.

## Conclusion

Study results indicate that school nurses need support in identifying mental illness among pupils. While school nurses felt responsible for their pupils and their well-being, they also felt that this responsibility should be shared by others working in the school, as well as guardians. A lack of specialist mental health education, clinical practice guidelines, and confidence were common themes that emerged from the interviews. The nurses’ own professional experience and desire to help were important but not sufficient. Providing extensive knowledge in the field of mental illness, in combination with training to enhance nurses’ capacity for critical thinking and the ability to carry out complex assessments, is thus recommended. Study results also indicate that there is a lack of research regarding the experiences of all professionals involved, which could contribute to a more comprehensive picture of the work required to identify mental illness among school pupils. As well, the results of the study suggest other research – for example, interventional studies that implement the guidelines and supports that the nurses in this study requested.
